# Impact of bronchoalveolar lavage from influenza A virus diseased pigs on neutrophil functions and growth of co-infecting pathogenic bacteria

**DOI:** 10.3389/fimmu.2024.1325269

**Published:** 2024-02-21

**Authors:** Simon Lassnig, Isabel Hennig-Pauka, Marta C. Bonilla, Matthias Mörgelin, Rabea Imker, Maren von Köckritz-Blickwede, Nicole de Buhr

**Affiliations:** ^1^ Institute of Biochemistry, University of Veterinary Medicine Hannover, Hannover, Germany; ^2^ Research Center for Emerging Infections and Zoonoses (RIZ), University of Veterinary Medicine Hannover, Hannover, Germany; ^3^ Clinic for Swine, Small Ruminants, Forensic Medicine and Ambulatory Service, University of Veterinary Medicine Hannover, Hannover, Germany; ^4^ Field Station for Epidemiology, University of Veterinary Medicine Hannover, Bakum, Germany; ^5^ Colzyx AB, Medicon Village, Lund, Sweden

**Keywords:** influenza A virus, *Pasteurellaceae*, neutrophil extracellular traps, pigs, co-infection, lung

## Abstract

**Introduction:**

Influenza A viruses (IAVs) infect the respiratory tract of mainly humans, poultry, and pigs. Co-infections with pathogenic lung bacteria are a common event and contribute to the severity of disease progression. Neutrophils are a major cell type of the innate immune system and are rapidly recruited to the site of infection. They have several effector functions to fight invading pathogens such as the secretion of reactive oxygen species (ROS) or the release of neutrophil extracellular traps (NETs). NETs are known to promote the growth of *Pasteurellaceae* bacteria, especially if degraded by nucleases.

**Methods:**

In this study, bronchoalveolar lavage fluid (BALF) from 45 field-infected pigs was analyzed for 1) NET markers, 2) influence on growth of lung bacteria, and 3) impact on neutrophil functions. BALF samples from 21 IAV-positive pigs and 24 lung diseased but IAV-negative pigs were compared.

**Results:**

Here, we show that neutrophils in the lungs of IAV-positive pigs release vesicular NETs. Several NET markers were increased in the BALF of IAV-positive pigs compared with the BALF from IAV-negative pigs. The amount of NET markers positively correlated with the viral load of the IAV infection. Interestingly, the BALF of IAV-positive pigs enhanced the growth of bacteria belonging to the family of Pasteurellaceae as potential coinfecting bacteria. These effects were weaker with the BALF derived from IAV-negative pigs with other lung infections. The intensity of oxidative burst in neutrophils was significantly decreased by BALF from IAVpositive pigs, indicating impaired antimicrobial activity of neutrophils. Finally, the lung milieu reflected by IAV-positive BALF does not enable neutrophils to kill *Actinobacillus pleuropneumoniae* but rather enhances its growth.

**Discussion:**

In summary, our data show that an IAV infection is affecting neutrophil functions, in particular the release of NETs and ROS. Furthermore, IAV infection seems to provide growth-enhancing factors for especially coinfecting Pasteurellaceae and reduces the killing efficiency of neutrophils.

## 1 Introduction

Influenza A virus (IAV) is a zoonotic pathogen that infects humans, pigs, and birds, causing respiratory disease with 300,000–600,000 human deaths annually (1).

During the H1N1 pandemic in 1918, approximately 95% of the fatalities were associated with bacterial co-infection (2, 3). Nowadays, co-infections are still a problem during influenza outbreaks and increase the risk for patients without further risk factors for intensive care unit admission (4).

In pigs, IAV usually causes severe illness with a relatively low mortality but can result in severe economic losses (5, 6). Bacterial co-infection is a common factor in disease complications (7, 8). Naturally occurring co-infections are poorly studied and surveilled due to costly diagnostics, but *in vivo* studies show increases in mortality with severe pneumonia (7–10).

Numerous mechanisms on how an infection with IAV raises the susceptibility of the host to a secondary bacterial infection are described, as well as the impairment of the innate immune system barriers, which enables bacteria to enter the tissue (11). Additionally, the virus interferes with the immune system and downregulates important cytokines that are needed for bacterial clearance or the recruitment and phagocytic behavior of neutrophils and alveolar macrophages (12–14).

Neutrophils are the most abundant cell type of the innate immune system with several effector functions. One is neutrophil extracellular trap (NET) formation. Upon activation, the DNA in the nucleus decondenses and is mixed with antimicrobial peptides. Outside of the cell, the DNA forms a web-like structure that entraps or kills pathogens. NETs can be induced by different stimuli, including pathogens like IAV (15–18). However, next to the beneficial effect of NETs for the host, detrimental effects have been identified in several studies with different diseases (19, 20). Interestingly, in IAV infection studies with mice, NETs were found in damaged lung areas, and therefore, a detrimental effect of NETs during IAV infection was discussed (21).

In previous studies, we have shown that human and porcine neutrophils are not able to control the growth of bacteria from the family *Pasteurellaceae* (22, 23). Rather, the degradation of NETs by DNases leads to the release of NET components such as NAD, promoting the growth of *Pasteurellaceae*. However, in pigs, the factors that initiate an infection with colonizing bacteria belonging to this family, including *Actinobacillus pleuropneumoniae [A. pleuropneumoniae (A.pp)]* and *Glaesserella* (*G.*) *parasuis*, are only poorly understood.

In this study, we show that during natural infection with IAV in pigs, vesicular NETs are induced in neutrophils that infiltrate the alveolar space. Furthermore, the cell-free supernatant of bronchoalveolar lavage fluid (BALF) of IAV-positive animals contains NET markers and sialic acid and thereby promotes the growth of lung pathogenic bacteria *in vitro*. On the other hand, the cell-free supernatant of BALF does not enable neutrophils to control an *A.pp* infection *in vitro*.

## 2 Materials and methods

### 2.1 Ethics statement

The handling and treatment of all animals were conducted in strict accordance with the principles outlined in the EU Directive 2010/63/EU and the German Animal Protection Law (Tierschutzgesetz). The blood sampling was approved by the authorities in the Committee on Animal Experiments of the Lower Saxonian State Office for Consumer Protection and Food Safety [Niedersächsisches Landesamt für Verbraucherschutz und Lebensmittelsicherheit (LAVES)], Lower Saxony, Germany, under the registration number: 33.9-42502-05-18A302.

The collection of BALF from diseased pigs sent for routine diagnostics was conducted in the Field Station for Epidemiology, University of Veterinary Medicine Hannover, Bakum, Germany. Pigs from swine farms in Lower Saxony, Germany, with visible symptoms of respiratory disease were euthanized for routine necropsy. The euthanasia was carried out according to the below-described “Guideline for the implementation of emergency killing of pigs.” The euthanasia of pigs due to other reasons, to collect BALF of healthy pigs, was approved and registered by the local Animal Welfare Officer in accordance with the German Animal Welfare Law under number TiHo-T-2019-14 (24).

### 2.2 Guideline for the implementation of emergency killing of pigs

Euthanasia of pigs as emergency killing of diseased and suffering pigs for diagnostic reasons was based legally on the Animal Welfare Act, Council Regulation (EC) No. 1099/2009 of 24th September 2009 on the protection of animals at the time of killing and the Animal Welfare Slaughter Ordinance of 1st January 2013 and was performed by skilled veterinarians.

In pigs with more than 5 kg of body weight, the skin was made wet. In the first step, pigs were anesthetized by electrical perfusion of the brain followed by immediate killing by cardiac perfusion of the heart [at least 1.3 A (sows 1.8–2 A) for 4 s]. For anesthesia, current-carrying forceps are placed at the ear bases, and current is conducted through the brain, leading to an epileptiform seizure accompanied by loss of perception and sensation. For killing, one electrode is then placed in the region of the heart and the other at the back at the level of the shoulder blades for ventricular fibrillation with subsequent cardiac arrest, which leads to the death of the animal.

Pigs with body weights below 5 kg and sows were euthanized by intravenous injection of pentobarbital in the *Vena cava cranialis* (piglets) or *Vena jugularis* (sows) using 45 mg of pentobarbital (Release^®^ 500 mg/ml, WDT Wirtschaftsgenossenschaft Deutscher Tierärzte eG, Garbsen, Germany) per kg body weight.

### 2.3 Euthanasia of healthy pigs

The pigs were anesthetized with azaperone [2 mg kg^−1^ body weight (BW), Stresnil ad us. vet., Elanco Tiergesundheit AG, Basel, Switzerland) and ketamine-hydrochloride (20 mg/kg BW, Ursotamin, 100 mg/ml, Serumwerk Bernburg AG, Bernburg, Germany) intramuscularly. The injection was carried out in the *Musculus biventer cervicis* near the base of the ear. The pig was immediately separated from the other pigs in the stable to allow a gentle start of the anesthesia. The depth of anesthesia was proven by observation of a trained veterinarian. Then, the pig was transported to a separate room for euthanasia. The pigs were euthanized intravenously via the auricular vein (*Vena auricularis*) with T 61^®^ (3–4 ml/50 kg BW, Intervet Deutschland GmbH, Unterschleißheim, Germany) during anesthesia. The death of the pig was determined by a trained veterinarian and confirmed by the absence of heartbeat and reflexes.

### 2.4 Obtaining bronchoalveolar lavage fluid

Immediately after euthanasia and opening of the carcass during necropsy, the lungs, trachea, and larynx were removed in total. The left main bronchus was clamped carefully behind the bifurcatio tracheae with an artery clamp before the lungs of the pigs were flushed via the glottis in portions of 100 ml four times with sterile phosphate buffered saline (PBS, in total 400 ml). Each portion of the BALF was harvested after gentle lung tissue massage via the glottis. All BALF portions were pooled and the volume of recovered fluid was recorded. The recovered BALF was spun down at 400×*g* and the supernatant was removed and frozen at −80°C. The cell pellet was resuspended in rest fluid and fixed in 4% paraformaldehyde after an aliquot was removed for influenza virus diagnostic by RT-PCR. Samples were used in the following assays freshly thawed on ice without any further processing.

### 2.5 Detection of IAV in respiratory diseased pigs

The presence of IAV was determined using the EZ-Universal Flu A 2.0 Real-Time RT-PCR Target-Specific Reagents for the Rapid Identification of IAV RNA from Tetracore. The BALF samples that tested positive for IAV were allocated to the group “IAV-positive,” and samples that tested negative were allocated to the group “IAV-negative.” Further important viral and bacterial pathogens of the swine were detected during routine diagnostics following the standard operating procedures of the accredited lab (European standard DIN EN IS0/IEC 17025). The results are shown in **Supplementary Table 1**.

### 2.6 Electron microscopy

Neutrophils present in the cell pellet of the BALF samples were fixed in PFA and were prepared for transmission electron microscopy (TEM) as previously published (25). The following antibodies were used: gold-labeled Anti-Histone H3 (citrulline R2 + R8 + R17) antibody (1:80 diluted; H3Cit, 5 nm gold; ab5103; Abcam, Berlin, Germany) and anti-neutrophil elastase (1:80 diluted; NE, 10 nm gold; ab131260; Abcam, Berlin, Germany).

### 2.7 ELISA analysis of the BALF supernatant

The following assays were conducted: the General Nicotinamide Adenine Dinucleotide (NAD) ELISA Kit (Ref: MBS2700640-96, MyBioSource, San Diego, California, UnitedStates), to detect the amount of NAD; the Citrullinated Histone H3 (Clone 11D3) ELISA Kit (Ref: 501620, Lot: 0637729, Cayman Chemical Company, Ann Arbor, Michigan, United States), to detect the amount of citrullinated histone H3 (H3Cit); the Cell Death Detection ELISA^PLUS^ Kit (Ref: 11774425001, Sigma), to detect the amount of nucleosomes; the Porcine IFN gamma (γ) ELISA Kit (Ref: KSC4021, Thermo Fisher Scientific Inc., Waltham, Massachusetts, United States), to detect IFN-γ; and the Porcine IFN alpha (α) ELISA Kit (Ref: ES7RB, Thermo Fisher Scientific Inc.), to detect IFN-α. The assays were performed according to the manufacturer’s recommendations. A sandwich ELISA for the detection of histone–MPO complexes was performed as described before (26).

### 2.8 PicoGreen assay

The amount of cell-free DNA in the BALF of pigs was evaluated by the Quant-iT™ PicoGreen™ dsDNA Assay-Kit (Thermo Fisher P11496). The analysis was performed according to the manufacturer’s protocol with some modifications. The PicoGreen was diluted 1:200 in Tris-EDTA buffer solution and then mixed 1:2 with the sample in a 96 black flat-bottom well plate (BRANDplates^®^ 781608) (100 μl final volume). A dilution series of the DNA standard of the kit was used for a standard curve. The fluorescence was measured in a TECAN Spark plate reader after a 5 min incubation period at room temperature in the dark.

### 2.9 DNase activity

DNase I Activity Assay Kit (BioVision, Milpitas, CA, USA, Fluorometric, K429-100) was used to determine the DNase I activity in the BALF samples. The test was performed following the manufacturer’s instructions.

### 2.10 Sialic acid detection

Sialic Acid Assay Kit (Ref: MAK314-1KT, Sigma-Aldrich) was used to determine free sialic acid (FSA) in the BALF samples. The test was performed following the manufacturer’s instructions.

### 2.11 Cultivation of *Actinobacillus pleuropneumoniae*

*Actinobacillus pleuropneumoniae* serotype (ST) 2 strain C3656/0271/11 was used in this study. This strain was isolated during routine diagnostics at the Institute of Microbiology, University of Veterinary Medicine Hannover, Germany, from the lung tissue of a diseased fattening pig during an *A.pp* outbreak (27). For the growth experiments, *A.pp* was grown on a fresh PPLO agar plate and incubated at 37°C in 5% CO_2_ overnight. For liquid cultures, Difco PPLO broth (Ref: 255420, BD, Franklin Lakes, New Jersey, United States) was used as recommended by the manufacturer. For the PMN killing assay, *A.pp* was cultivated as previously described (23). The supplement solution (IsoVitaleX) needed for the optimal growth of *A.pp* and *G. parasuis* was prepared as described previously (22). IsoVitaleX enrichment supplies the V-factor (nicotinamide adenine dinucleotide, NAD) and chemically defines the substances for the cultivation of nutritionally fastidious microorganisms.

### 2.12 Cultivation of *Glaesserella parasuis*

*Glaesserella parasuis* serotype 13, isolated from a diagnostic sample from a diseased pig with pneumonia (lung; lab number 21/981), was freshly grown on Columbia agar with chocolated horse blood plates (Oxoid, Deutschland GmbH, Wesel, Germany Ref: PB0124A) at 37°C in 5% CO_2_ overnight. For liquid cultures, Difco PPLO broth (Ref: 255420, BD, Franklin Lakes, New Jersey, United States) was used as recommended by the manufacturer, supplemented with 1% Tween 80 (10% in ddH_2_O) and 1% IsoVitaleX substitute.

### 2.13 Cultivation of *Streptococcus suis*

*Streptococcus* (*S.*) *suis* strain 385, isolated from the lung of a diseased pig, was used in the experiments and was freshly grown on Columbia blood agar plates with 7% sheep blood from Oxoid (Ref: PB5008A) at 37°C overnight. For liquid cultures, Todd Hewitt broth (Ref: 249450, BD) was used as recommended by the manufacturer.

### 2.14 Bacterial growth in the presence of porcine BALF

In each well of transparent 96-well flat-bottom plates, 60 µl of PPLO medium was spiked with 130 µl (for *A.pp*) of porcine BALF supernatant of eight IAV-positive and six IAV-negative animals. The 14 used samples were selected from the 45 BALF samples because they were declared by routine microbiological examination as free from any detected bacterial or viral contamination (see **Table S1**). All BALF samples were cell-free supernatant and had been sterile-filtered before. Equal volumes of PPLO and PBS served as a control. *A.pp* was grown as described above, and a sufficient amount of colony material was transferred into sterile, LPS-free 1X PBS and an OD at *λ* = 600 nm of 0.5 was adjusted. Ten microliters of this bacterial suspension was added to each well, and the plate was incubated at 37°C in 5% CO_2_ in the Tecan Spark plate incubator. The optical density was measured at 600 nm every 15 min for 6 h. To determine the slope of the growth curve, the blank (OD of *t*_0_) was subtracted and the mean value of the triplicates was calculated. The mean values of all BALF samples were pooled in the IAV-positive and IAV-negative groups, and a simple linear regression was calculated. The slope of three technical replicates was used to calculate the statistics.

For *S. suis* and *G. parasuis*, the assay was performed as described above, with minor changes. For *S. suis*, 50 µl of BALF was added to 140 µl of THB medium, and incubation was performed at 37°C in 0% CO_2_. *G. parasuis* with 170 µl of BALF and 20 µl of PPLO medium supplemented with 1% Tween 80 solution (10%) and 1% IsoVitaleX substitute was incubated at 37°C in 5% CO_2_. Adding the bacteria resulted in 200 µl of liquid culture.

### 2.15 Assay to detect reactive oxygen species production and cytotoxic effects in porcine neutrophils using flow cytometry

Intracellular reactive oxygen species (ROS) production was measured using 2′,7′-dichlorodihydrofluorescein diacetate (DCFH-DA) (Invitrogen, Carlsbad, CA, USA). To measure cell death, propidium iodide (PI) (Sigma-Aldrich, St. Louis, USA) was used. The method used was previously published by Bengoa et al. (28). Here, freshly isolated porcine neutrophils [2 × 10^5^ dissolved in 75 µl of Roswell Park Memorial Institute (RPMI) 1640 medium] were seeded in a 96-well transparent, flat-bottom plate. The cells were stimulated with 100 µl of BALF. The included BALF samples are the same as those used in the growth experiments (eight sterile filtered IAV-positive and six IAV-negative BALF samples).

Cells incubated only in RPMI and without added dye served as an unstimulated control. Cells incubated with 25 nM of phorbol-12-myristate-13-acetate (PMA) (Sigma-Aldrich) served as a positive control for ROS production. Heat-killed neutrophils (10 min, 70°C) and a 50:50 mixture of live and heat-killed cells were used as a positive control for cell death.

To each sample, except the unstimulated control, 10 µM of DCFH-DA and 0.25 µg/ml of PI in 25 µl RPMI 1640 were added (RPMI only for unstained controls), and the plates were incubated for 1 h and 3 h (37°C, 5% CO_2_), respectively.

The ROS and PI levels were measured immediately after incubation using the Attune NxT Cytometer (Thermo Fisher). Acquisition volume was set to 50 µl and acquisition speed was set to 100 µl/min. FSC and SSC settings were optimally adjusted to the size and granularity of neutrophil granulocytes. ROS was detected with the BL1 530/30 filter of the blue laser (488 nm) and PI with the YL1 585/16 filter of the yellow laser (561 nm).

Data were analyzed with FlowJo™ 10.8.1 software (Ashland, OR, USA). The gating strategy included only singlets of the neutrophil population. The threshold for ROS-positive cells was set using the unstimulated and unstained cells. Dead cells were determined using heat-killed cells as a threshold. In each technical run, duplicates were analyzed. In total, three independent experiments were conducted, using three different blood donors.

### 2.16 Purification of porcine neutrophils

Porcine neutrophils were purified using BioColl (1.077 g/ml; Bio&SELL, Feucht, Germany #BS.6556381.5) and hypotonic lysis of erythrocytes as described previously (29). Cells were resuspended in RPMI 1640 (without phenol red, PAA Laboratories, Inc. Pasching Austria).

### 2.17 Neutrophil killing assay with *Actinobacillus pleuropneumoniae* and porcine BALF

In each well of 48-well suspension plates (Greiner Bio-One, 677102, Kremsmünster, Austria), 2 × 10^5^/100 μl porcine neutrophils were seeded and infected with *A.pp* ST 2 (MOI = 2). Samples were incubated with and without 50 µl of sterile filtered BALF of one IAV-positive and one IAV-negative pig. RPMI medium was added to complete the volume of each well to 200 μl. The plates were centrifuged (370×*g*, 5 min) and incubated for 1 h at 37°C in 5% CO_2_.

To determine the CFU/ml, a serial dilution at time points 0 h and 1 h was plated on PPLO agar plates and incubated for approximately 17 h at 37°C in 5% CO_2_. The survival factor (SF) was calculated with the formula SF_1h_ = CFU_1h_/CFU_0h_. The ratio of BALF-IAV-positive and BALF-IAV-negative was calculated with the formula ratio = (SF neutrophils + BALF)/(SF neutrophils).

The IAV-negative BALF sample was chosen by the parameter of having no detected bacterial or viral infection. The IAV-positive sample with the lowest Ct value in the lung and BALF was chosen from the samples without bacterial or other viral infections.

### 2.18 Statistics

Data were analyzed using Excel 2016 (Microsoft) and GraphPad Prism version 10.0.2 (232) (GraphPad Software, San Diego, CA, USA). Normal distribution was tested using the D’Agostino & Pearson K2 normality test (GraphPad Software, San Diego, CA, USA). Differences and correlations between groups were tested as indicated in the figure legends (*p < 0.05, **p < 0.01, ***p < 0.001).

## 3 Results

### 3.1 Porcine neutrophils of pigs naturally infected with IAV release vesicular NETs

**Figure 1:**
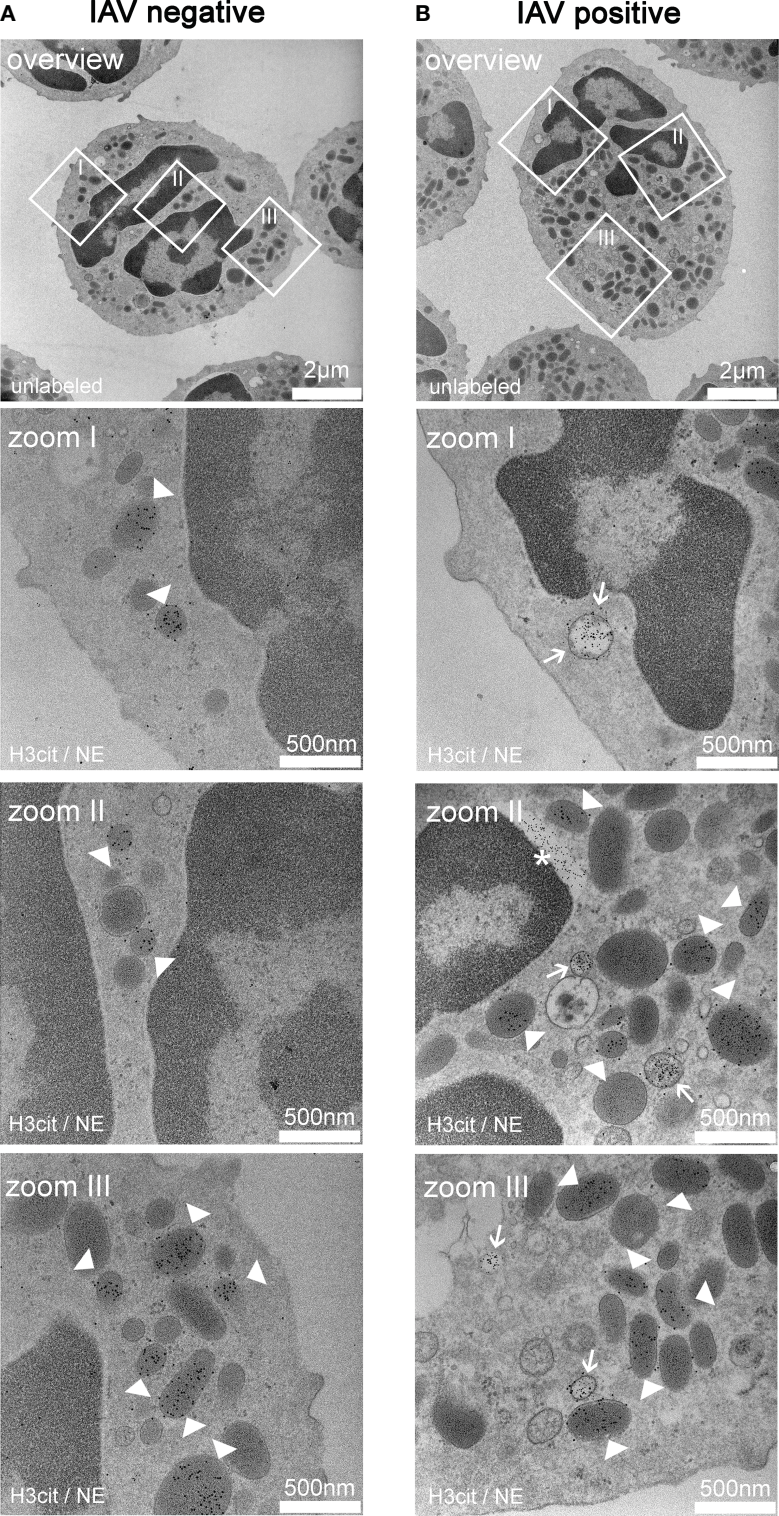
Exemplary TEM images derived from neutrophils from the bronchoalveolar lavage fluid (BALF) of respiratory diseased pigs. **(A)** Pictures of a neutrophil of a pig tested negative for influenza A virus (IAV). The pictures (zoom I–III) show vesicles containing neutrophil elastase (NE) (white arrowheads) in the cytoplasm. The white squares mark the zoomed area. **(B)** Pictures of a neutrophil of a pig tested positive for IAV. The pictures (zoom I–III) show the colocalization of NE and H3cit in the vesicles (white arrow) in the cytoplasm and vesicles containing NE alone (white arrowheads). Extracellular H3cit signal and blebbing of the nuclear membrane is labeled with an asterisk (*). The white squares in the overview mark the zoomed area. **(A, B)** 5 nm of gold-labeling H3Cit; 10 nm of gold-labeling NE.

As an initial starting point of this study, we analyzed by TEM if porcine neutrophils release NETs *in vivo* inside the alveolar space during a natural IAV infection (**Figure 1**). In neutrophils from an IAV-negative pig, only NE-positive granules and no pronounced NET vesicles were detected (**Figure 1A**). On the other hand, neutrophils collected from the BALF of an IAV-positive pig had neutrophil elastase (NE) and H3Cit-positive vesicles in the cytoplasm (**Figure 1B**). NE is a key enzyme of neutrophils and H3Cit is a product of the protein arginine deiminase 4 (PAD4), involved in NET formation. Both are involved in the decondensation of chromatin and markers for NET formation (30). As NET marker-positive vesicles are formed and the outer neutrophil membrane is intact, vesicular NET formation was detected in neutrophils from the BALF of an IAV-positive pig.

### 3.2 NET markers are present in the BALF of influenza diseased pigs and correlate with the IAV load

Next, we analyzed the BALF samples of 50 pigs (45 were naturally infected with respiratory diseases and showed clinical symptoms, and five animals were healthy controls). Twenty-one of 45 diseased animals were tested positive for IAV (**Table S1**). A bacterial infection was detected in 30 of 45 pigs, and a bacterial co-infection occurred in 12 of the IAV-positive animals. In five pigs, other porcine viruses were detected.

**Figure 2:**
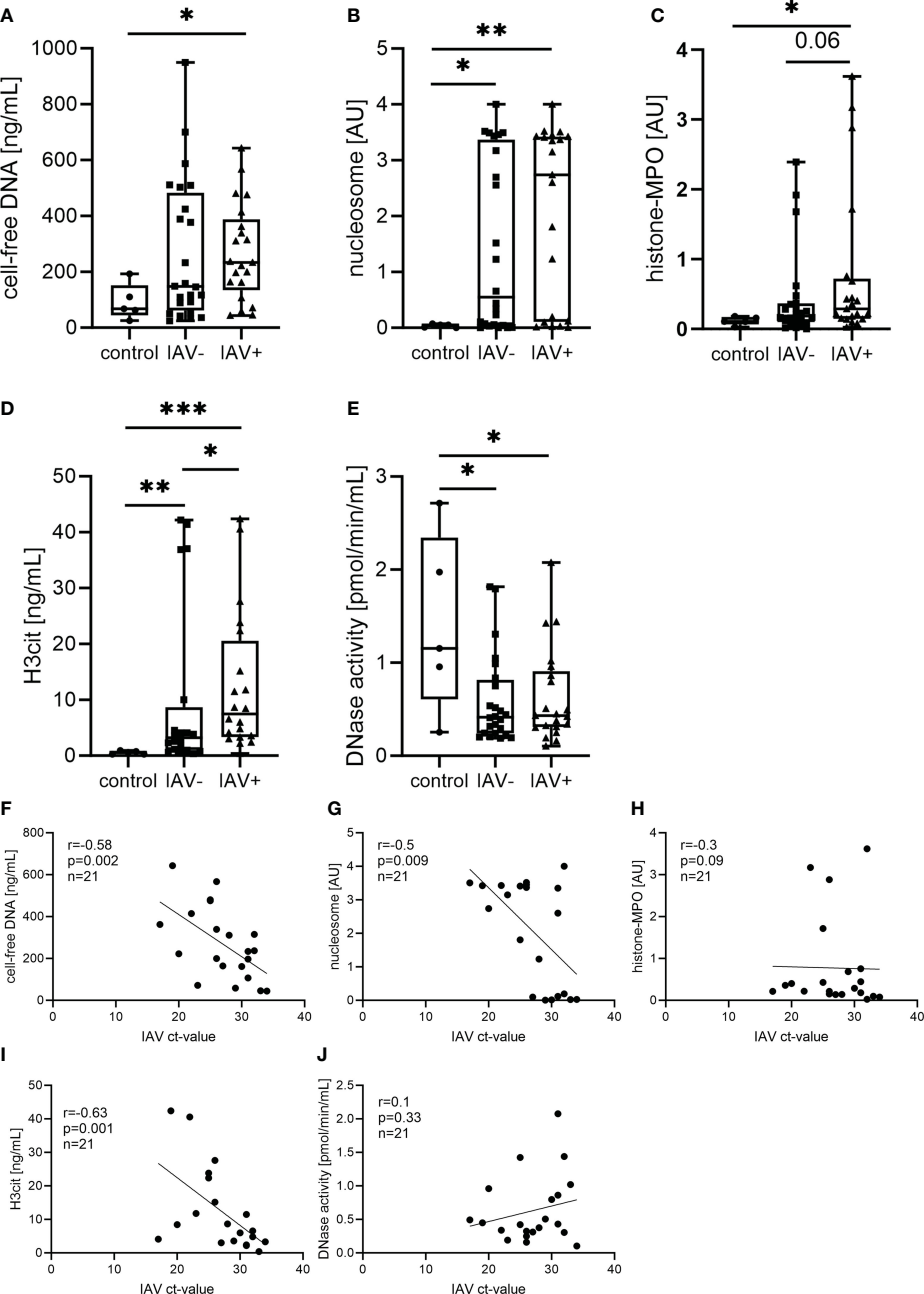
Neutrophil extracellular trap (NET) markers in the BALF of respiratory diseased pigs tested negative (IAV-negative) and positive (IAV-positive) for IAV **(A–E)** and the correlation to the Ct value detected in BALF and/or lung tissue **(F–J)**. The amounts of cell-free DNA **(A)**, nucleosomes **(B)**, histone–myeloperoxidase (MPO) complexes **(C)**, citrullinated histone-3 (H3Cit) **(D)**, and DNase I activity **(E)** were analyzed in the BALF of 21 IAV-positive, 24 IAV-negative, and five (four in DNase I) healthy control animals. For each marker, a box and whisker plot with the minimum and maximum values and individual points is presented. A Mann–Whitney test was calculated (*p < 0.05, **p < 0.01, ***p < 0.001). The correlation of the NET marker and the Ct value of IAV was performed with Spearman *r* resulting in a significant negative correlation of cell-free DNA **(G)**, nucleosomes **(H)**, and H3Cit **(J)**. A simple linear regression is depicted in the graph and the values for *r*, *p*, and *n* are given.

NET markers were quantified in BALF (**Figure 2**). The amount of cell-free DNA was measured, as NETs consist of a DNA backbone. A significant difference was detected between IAV-positive and control pigs (**Figure 2A**). Nucleosomes can be measured extracellularly upon NET formation (31). Significantly higher amounts of nucleosomes were detected in the BALF of infected pigs compared with uninfected pigs, but no difference between IAV-positive and IAV-negative pigs was measured (**Figure 2B**). Myeloperoxidase (MPO) is one of the main antimicrobial components that can be found attached to NETs. In the form of histone–MPO complexes, it serves as a very specific NET marker as these complexes are not formed under necrotic circumstances (26, 32). Significantly higher amounts of histone–MPO complexes were detected in IAV-positive BALF compared with controls (**Figure 2C**). Finally, we measured the levels of H3Cit. This citrullination is mediated by PAD4 and promotes the decondensation of chromatin during the formation of NETs (25, 30, 33, 34). The diseased groups (IAV-positive and IAV-negative) had significantly increased amounts of H3Cit compared with the controls. Furthermore, significantly more H3Cit was measured in the IAV-positive group compared with the IAV-negative group (**Figure 2D**).

Therefore, NET formation takes place during an IAV infection inside the lungs in pigs. To prevent the detrimental effects of NETs, the host releases DNase to degrade NETs (19). DNase activity was measured as an indirect NET marker. However, DNase activity was significantly reduced in both infected groups compared with the control (**Figure 2E**).

As we have found increased amounts of several NET markers in the IAV-positive BALF samples, we were interested in finding out if there was a correlation to the severity of the influenza-related lung disease. As a marker of severity, we used the Ct values of the IAV RT-PCR in BALF pellets and/or lung tissue. A significant negative correlation of the IAV Ct value with the amounts of H3Cit, cell-free DNA, and nucleosomes was found in the BALF of IAV-positive pigs (**Figures 2F, G, I**). The levels of DNase activity and histone–MPO complexes show no correlation with the severity of infection (**Figures 2H, J**).

### 3.3 Sialic acid is present in the BALF of influenza diseased pigs

**Figure 3:**
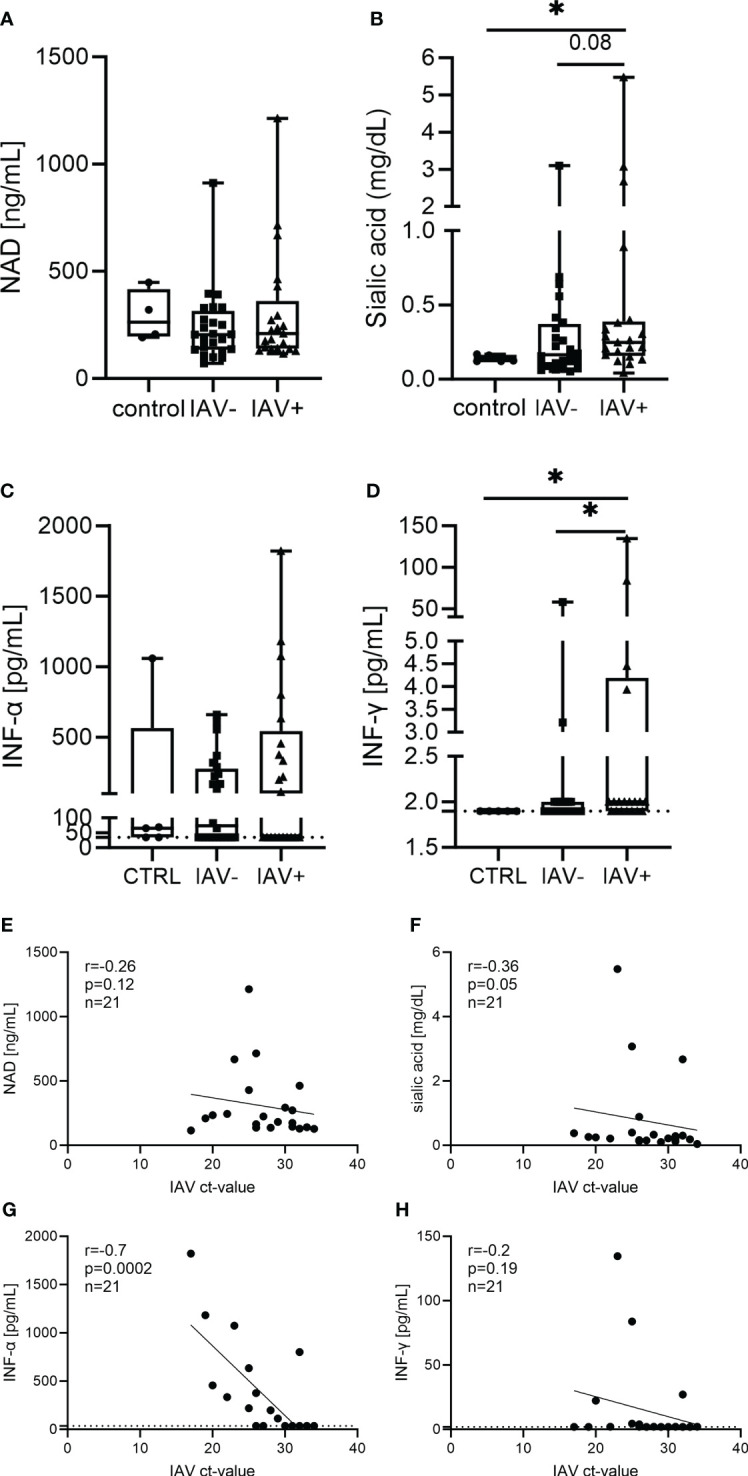
Different markers in the BALF of respiratory diseased pigs tested negative (IAV-negative) and positive (IAV-positive) for IAV **(A–D)** and the correlation to the IAV Ct value detected in BALF and/or lung tissue **(E–H)**. The amounts of nicotinamide adenine dinucleotide (NAD) **(A)**, sialic acid **(B)**, IFN-α **(C)**, and IFN-γ **(D)** were analyzed in the BALF of 21 IAV-positive, 24 IAV-negative, and five (four in NAD) healthy control animals. For each marker, a box and whisker plot with the minimum and maximum values and individual points is presented. A Mann–Whitney test was calculated (*p < 0.05). The correlation of the analyzed markers and the Ct value of IAV was performed with Spearman *r* resulting in no significant correlation, except for a significant negative correlation between IAV Ct value and IFN-α **(G)**. A simple linear regression is depicted in the graph and the values for *r*, *p*, and n are given **(E–H)**. The dotted line **(C, D, G, H)** represents the detection limit of the ELISA for IFN-α (36 pg/ml) and IFN-γ (2 pg/ml), as all the samples where no signal was detected were set to the detection limit.

Next, we further analyzed markers in the BALF that could be increased by IAV infection and NET formation (**Figure 3**). In a previous study, we identified NAD to be present in the supernatant of NETs, and it was released upon degradation with DNase (23). As NAD is a growth factor for *Pasteurellaceae*, we measured its levels in the BALF; however, no difference was detectable (**Figure 3A**). As an IAV infection increases the sialic acid content, we determined free sialic acid in BALF. Significantly higher amounts of sialic acid were detected in the BALF of IAV-positive pigs compared with healthy controls. However, there was no significant difference in IAV-negative but lung disease pigs detected (**Figure 3B**). As interferons (IFNs) are strongly induced by IAV, finally, we measured IFN-α and IFN-γ in BALF. When measuring IFN-α, approximately half of the samples in each group (23 of 51 samples in total) did not show a signal higher than the blank value. These samples are below the detection limit of 36 pg/ml. Even the highest values were detected in the IAV-positive BALF, and no significant difference between the groups was observed. In the IFN-γ measurement, only six samples were clearly positive for IFN-γ (two IAV-negative and four IAV-positive BALF samples) (**Figure 3D**). All the other samples were either close to or below the detection limit of the ELISA (2 pg/ml).

The levels of NAD and IFN-γ show no correlation with the severity of infection (**Figures 3E, H**). A significant negative correlation of the IAV Ct value with the amount of sialic acid and IFN-α was found in the BALF of IAV-positive pigs (**Figures 3F, G**).

### 3.4 The BALF of influenza diseased pigs enhances the growth of lung pathogenic bacteria

**Figure 4:**
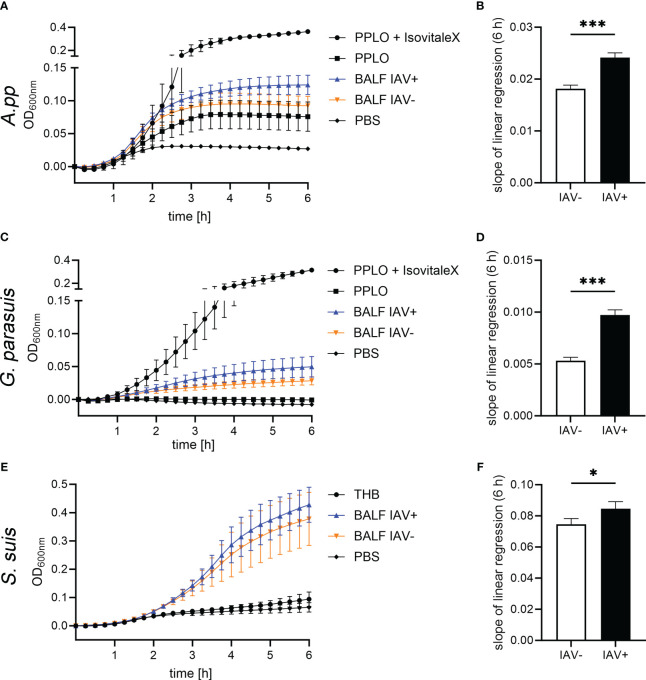
The BALF samples of IAV-positive animals significantly enhance the growth of bacterial lung pathogens. The amount of BALF added to the medium was determined in a previous experiment. **(A, C, E)** These growth curves show the growth of *Actinobacillus pleuropneumoniae (A.pp)*, *G. parasuis*, and *S. suis* in the first 6 h in the PPLO medium spiked with the BALF of IAV-positive or IAV-negative pigs or PBS. For *G. parasuis* and *A.pp*, the PPLO medium with and without the IsoVitaleX substitute served as a control; for *S. suis*, the THB medium was used. Data are presented as mean ± SEM. **(B, D, F)** The slope of the linear regression performed on the growth curves that were spiked with IAV-positive and IAV-negative BALF is depicted in this diagram. *A.pp*, *G. parasuis*, and *S. suis* show a significantly increased growth in the presence of IAV-positive BALF compared with IAV-negative BALF. Data were analyzed with one-tailed paired Student’s *t*-test (*p < 0.05, ***p < 0.001) and are presented as mean ± SD (*n* = 3 experiments with the mean of triplicates of eight IAV-positive and six IAV-negative BALF samples are presented).

After we quantified NET markers and other markers in BALF, we investigated if BALF has an effect on the growth of the pathogenic lung bacteria *A.pp*, *G. parasuis*, and *S. suis* (**Figure 4**). The bacteria were grown in their respective growth media, supplemented with BALF. The BALF samples of eight IAV-positive animals and six IAV-negative animals without a diagnosis of additional bacterial or viral contamination were included. We detected a significantly steeper slope of the growth curves in the bacteria grown with IAV-positive BALF, indicating a faster growth and finally a higher number of resulting bacteria (**Figures 4B, D, F**). The growth-enhancing effect in *A.pp* (*p* = 0.0005) and *G. parasuis* (*p* = 0.0001) was higher than in *S. suis* (*p* = 0.02). Therefore, the BALF of IAV-positive pigs leads to enhanced growth of possible coinfecting agents, especially if they belong to the *Pasteurellaceae*.

### 3.5 Several factors in the BALF of IAV-positive animals are potential growth factors for *Actinobacillus pleuropneumoniae*

**Figure 5:**
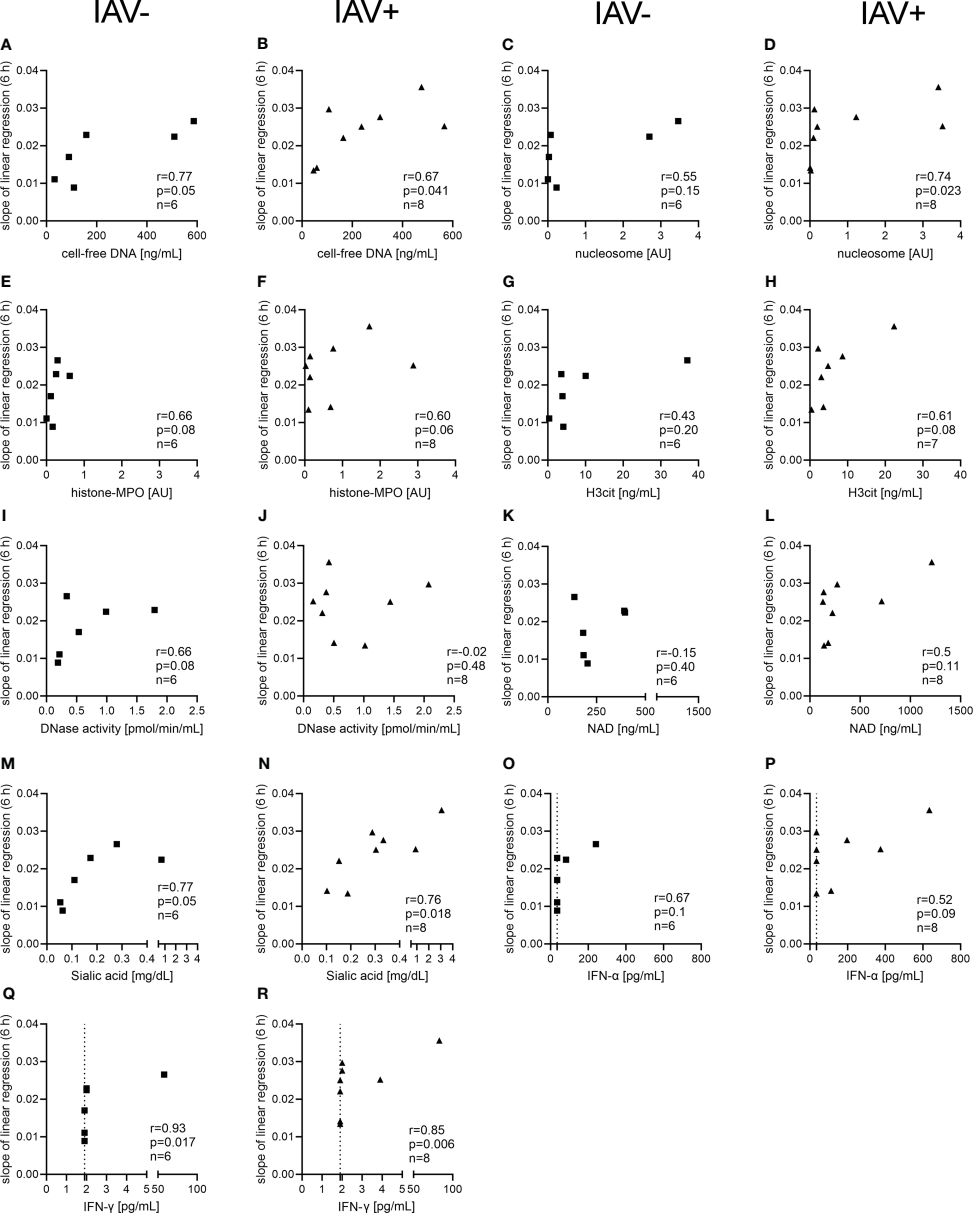
Correlation analysis of NET markers and other markers detected in the BALF with the growth progression of *A.pp* in the BALF. The correlation analysis was performed with Spearman *r* for IAV-negative BALF **(A, C, E, G, I, K, M, O, Q)** and for IAV-positive BALF **(B, D, F, H, J, L, N, P, R)** and detected several significant positive correlations. The growth progression of *A.pp* correlates positively with cell-free DNA **(A, B)**, sialic acid **(M, N)**, and IFN-γ **(Q, R)** in the case of IAV-negative and IAV-positive BALF. The growth progression of *A.pp* correlates with the nucleosome **(D)** only in the case of IAV-positive BALF. The values for *r*, *p*, and *n* are given in each graph, and each square or triangle represents one BALF sample. Several markers in the BALF correlate positively with the growth progression of *A.pp*, but this was not significant. The dotted line **(O–R)** represents the detection limit of the ELISA for IFN-α (36 pg/ml) and IFN-γ (2 pg/ml), as all the samples where no signal was detected were set to the detection limit.

As we detected several factors in the BALF from IAV-positive animals significantly increased (**Figures 2**, **3**) and a bacterial growth-enhancing effect of BALF from IAV-positive animals, we wondered if some of the detected factors could explain the phenotype. As the strongest growth-enhancing effect of the IAV-positive BALF compared with IAV-negative BALF was seen in *A.pp* growth experiments, we conducted a correlation analysis for the growth progression of *A.pp* (**Figure 5**). Cell-free DNA, sialic acid, and IFN-γ significantly correlate with the growth progression in IAV-negative and IAV-positive BALF (**Figures 5A–D, M, N**). The nucleosome significantly correlates with the growth progression only in IAV-positive BALF (**Figure 5D**). Furthermore, some factors (histone–MPO, H3Cit, and DNase activity) correlate positively with the growth progression of *A.pp* in BALF, but narrowly missed significance (**Figures 5E, F, H, I**). Therefore, several potential growth-enhancing factors for *A.pp* are detected in the BALF of infected animals but not exclusively in IAV-positive animals.

### 3.6 The BALF of influenza diseased pigs significantly inhibits respiratory burst in porcine neutrophils

**Figure 6:**
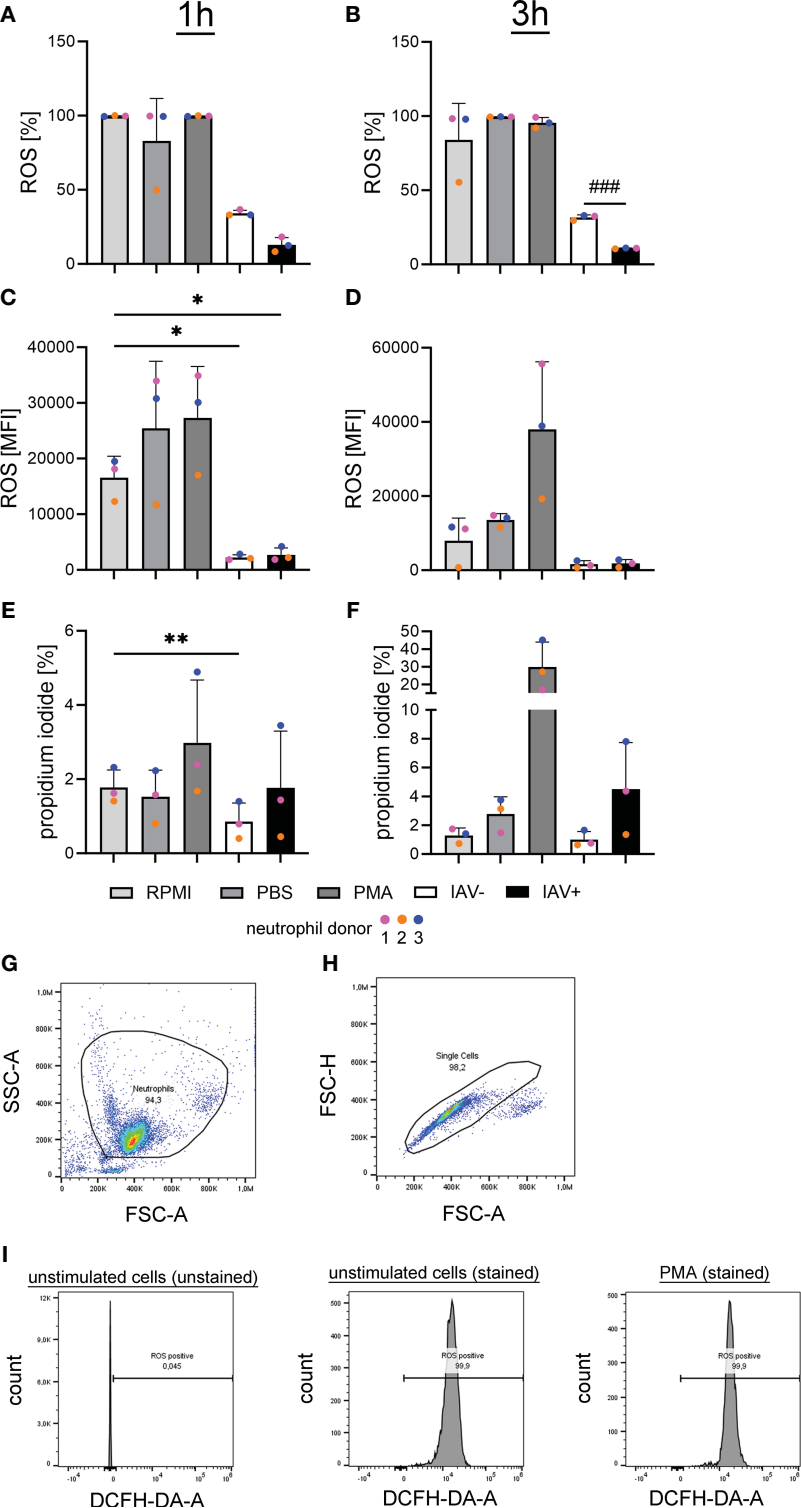
Oxidative burst in freshly isolated porcine neutrophils is significantly reduced after incubation with the BALF of IAV-positive or IAV-negative pigs. RPMI was used as a negative control and phorbol-12-myristate-13-acetate (PMA) stimulation as a positive control. BALF has no cytotoxic effects on freshly isolated porcine neutrophils **(A–F)**. **(A, B)** The intracellular ROS production was determined by adding 2′-7′-Dichlorodihydrofluorescein diacetate (DCFH-DA) to neutrophils after stimulation with the BALF samples, RPMI, PBS, or PMA. A significant decrease in ROS-releasing cells after 3 h **(B)** was detected comparing neutrophils incubated with the BALF of IAV-positive and IAV-negative animals. **(C, D)** The MFI of ROS-positive cells is presented and a significant decrease was observed after treatment with IAV-positive BALF and IAV-negative BALF after 1 h **(C)** of incubation compared with incubation with RPMI. **(E, F)** The amount of dead neutrophils in percent after staining with PI is presented, and a significant decrease in cytotoxicity is detected in BALF IAV-negative compared with RPMI after 1 h of incubation **(E)**. Data were analyzed with one-way ANOVA (*p < 0.05, **p < 0.01) and with one-tailed paired Student’s *t*-test (###*p* < 0.001) and are presented as mean ± SD (*n* = 3 experiments with the mean of duplicates of eight IAV-positive and six IAV-negative BALF samples are presented). **(G–I)** The gating strategy for the analysis of DCF-positive cells [oxidation of DCFH-DA by ROS results in fluorescence of 2′-7′-dichlorofluorescein (DCF)] by flow cytometry is presented. **(G)** Based on FSC-A and SSC-A, the neutrophil population was gated. **(H)** Based on FSC-A and FSC-H, all singlets were gated from the neutrophil population. **(I)** The threshold for ROS-positive cells was adjusted in the population of unstimulated and unstained cells. Example histograms of different samples are presented. The gating of propidium iodide-positive cells was performed in a similar manner with the same cell subset in the channel “PI-A.” Each colored dot in **(A)** represents the results in the different groups from one experiment (= one neutrophil donor).

An infection with IAV can promote bacterial co-infections in multiple ways. Next to the detected direct growth-enhancing effect on bacteria, we investigated whether BALF acts on the host’s innate immune system. To test the effect of the milieu during an IAV infection on neutrophils, we measured ROS upon stimulation with BALF. The formation of ROS is an important effector function of neutrophils and a marker for neutrophil activation. The incubation with BALF from IAV-positive animals resulted in a significant decrease in the amount of ROS-producing cells compared with the BALF from IAV-negative animals (**Figure 6**). Additionally, the incubation with the BALF of diseased animals in general caused a significant inhibition of ROS compared with the PBS control. The mean fluorescence intensity (MFI), the amount of ROS that is produced by each ROS-producing cell, was also significantly reduced after incubation with BALF compared with control cells in RPMI.

These data show that the antimicrobial effect of neutrophils by ROS formation is impaired by BALF derived from diseased animals. To see if this effect is due to neutrophil death or ROS-inhibiting effects, the survival of neutrophils was measured.

Using PI staining of neutrophils and subsequent flow cytometry, we did not see an increase but rather a significant decrease in the PI staining in neutrophils treated with BALF from IAV-negative animals (**Figures 6E, F**). These data suggest that factors released during lung infections severely inhibit the respiratory burst of the neutrophils. The presence of IAV seems to increase this phenotype.

### 3.7 Neutrophils are unable to control *Actinobacillus pleuropneumoniae* growth in the presence of BALF from IAV-positive pigs

With the newly gained insight that the BALF of IAV-positive animals is on the one hand enhancing the growth of *Pasteurellaceae* bacteria and on the other hand inhibiting the respiratory burst of neutrophils, we investigated how BALF affects the antimicrobial effect of neutrophils. By adding BALF, which reflects the lung milieu in the form of diluted epithelial lining fluid covering the airways, an *in vivo*-mimicking environment was created. In a final step, we analyzed whether this environment supports or diminishes the killing capacity of neutrophils from *A.pp*.

**Figure 7:**
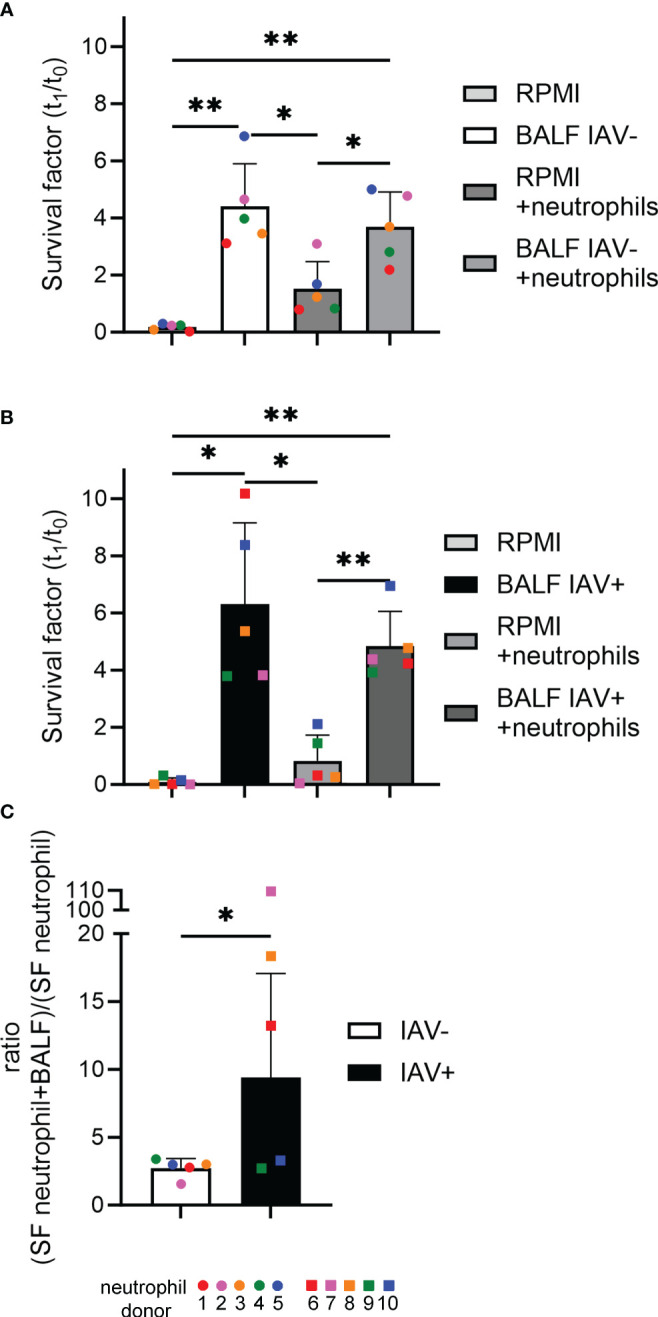
The BALF of IAV-positive animals significantly increases the amount of bacteria surviving in the presence of neutrophils and NETs. **(A, B)** *A.pp* was grown for 1 h at 37°C in 5% CO_2_ in the absence or presence of neutrophils from 10 different donors and the BALF of an IAV-positive or IAV-negative animal. The survival factor (SF) was calculated [*n* = 5 in **(A)** and *n* = 5 in **(B)**]. **(C)** The ratio of the SF of the samples “RPMI+neutrophils” and “BALF (IAV-positive or IAV-negative) + neutrophils” was calculated. All data are presented with mean ± SD and were analyzed with one-tailed paired Student’s *t*-test and one-way ANOVA (*p < 0.05, **p < 0.01. Each colored dot in **(A)** represents the results in the different groups from one experiment (= one neutrophil donor). Each colored square in **(B)** represents the results in the different groups from one experiment (= one neutrophil donor). Each colored dot (IAV-negative) or colored square (IAV-positive) in **(C)** represents the respective calculated ratio from **(A)** or (**B**).

The incubation of *A.pp* in combination with BALF resulted in a significantly higher survival factor (SF = 6.3 for IAV-positive, SF = 4.4 for IAV-negative) of *A.pp* (**Figure 7**). This goes in line with the results of the growth experiments (**Figure 4**). The SF of the bacteria incubated together with the neutrophils (RPMI+neutrophils) ranges around 1, which means the bacterial number is constant, indicating that the bacteria are not being killed by the neutrophils. The combination of *A.pp*, neutrophils, and BALF resulted in an increased SF. This phenotype occurs in IAV-positive and IAV-negative samples in a comparable tendency. However, as the assays were conducted with different donor pigs, this can influence the result. By calculating the ratio of the SF between RPMI+neutrophils and BALF+neutrophils, a significant difference was identified between IAV-positive and IAV-negative BALF, indicating that the BALF of IAV-positive animals increases the survival of *A.pp* even stronger (**Figure 7C**).

## 4 Discussion

In this study, we addressed the question of whether an infection with IAV stimulates neutrophils *in vivo* to produce NETs and whether these NETs play a role in secondary infections with lung pathogenic bacteria.

Neutrophils inside the alveolar space of an IAV-infected pig had an intact cellular membrane of neutrophils, and vesicles filled with NE and H3cit were in the cytoplasm (**Figure 1B**). These characteristics were first described for vesicular NET formation in neutrophils infected with *Staphylococcus aureus* or *Candida albicans* (35, 36). To the best of our knowledge, we identified for the first time vesicular NET formation during IAV infection. In contrast to suicidal NETosis, the pathway of vesicular NETosis results in a more controlled NET formation. Chromatin is transported to the outer membrane in vesicles, leaving the cell intact and viable. This pathway which is also called vital NETosis allows the neutrophil to perform effector functions after the release of NETs (37, 38). Vesicular NETosis is described to work independently of NADPH oxidase (36). This goes in line with the observed significant decrease in ROS-producing cells and intensity of respiratory burst when the neutrophils were treated with the BALF of IAV-infected animals (**Figure 6**) (36). It still remains to be determined if suicidal NET formation is the consequence of a later stage of IAV-induced neutrophil activation and can be detected besides vesicular NET formation in pigs. In mice, suicidal NETs in reaction to IAV have been shown (21, 39). Furthermore, we aimed to detect the potential cytotoxic effects of BALF components on freshly isolated neutrophils. However, no increase in dead cells was detected compared with controls (**Figure 6**). Small numbers of dead cells in combination with evidence for NET formation can be a hint for vesicular NET formation. Bengoa et al. detected NET levels in human neutrophils at approximately 50% together with dead cells below 5% and confirmed vesicular NET formation with electron microscopy analysis (28). It needs to be considered that we have measured dead cells only in isolated neutrophils stimulated with BALF instead of in the *in vivo*-infected neutrophils, where we detected vesicular NET formation. Therefore, further *in vivo* samples are needed to prove this hypothesis.

In addition to the detection of vesicular NET formation, several NET markers were quantified in the BALF samples. The increased levels of cell-free DNA, nucleosomes, H3Cit, and histone–MPO complexes compared with the control animals show that NET formation is present in respiratory lung diseases. In this project, we used field samples that contained a broad variety of infectious agents (**Table S1**), which can be made responsible for NET induction. The presence of NET markers in the group of IAV-negative animals is therefore not surprising and can be caused by a variety of coinfecting pathogens (15, 22, 23, 40, 41). The lack of correlation of the histone–MPO complexes with the virus burden raised the assumption that necrotic effects can at least partially influence the detection. Cell-free DNA and nucleosomes are considered NET markers, which are correlated to the IAV burden; however, their presence can be caused by necrosis (42, 43). Hayden et al. reported the lack of specificity of DNA–MPO ELISAs, which failed to correctly detect DNA–MPO complexes in *in vivo* plasma samples. It showed a lack of correlation to disease severity (44). In our study, however, we have used an ELISA to detect histone–MPO complexes. These are considered as more specific to NET formation as DNA–MPO complexes, as they do not form naturally (26, 32). Additionally, our assay used a different antibody set. However, we have tested for another specific NET marker, H3Cit, which was increased in the BALF and correlated to the severity of IAV (**Figure 2I**) (25, 30, 33, 34). Therefore, it needs to be considered that for studying NETs *in vivo*, a combined analysis of more than one NET marker is needed.

Interestingly, a significant correlation between IAV burden and the increase of some of the tested NET marker levels in BALF was observed (**Figures 2F, G, I**). This result is supported by Zhu et al., who found correlations between NET levels in the plasma and the severity of an IAV infection in mice. Additionally, they have shown that IAV-induced NETs cause lung epithelial damage (45). By the destruction of lung tissue, IAV is increasing the risk for bacterial co-infections. Narasaraju et al. showed with IAV infection studies in mice that NETs lead to acute lung damage. Excessive NET formation induced detrimental effects on endothelial and epithelial tissues in the lung, worsening the disease (21, 45). Therefore, the IAV-induced NETs weaken the host for possible bacterial co-infections. This can become even worse if the co-infection involves bacteria that are not efficiently killed by NETs. On the one hand, this includes bacteria from the family of *Pasteurellaceae* that can use NET components as growth factor (22, 23). On the other hand, this includes bacteria that produce DNases to escape out of NETs like *Streptococcus suis* in pigs or *Streptococcus pneumoniae* in humans as they can use NETs for spreading (46–48). All of these bacteria are potential co-infection pathogens in the lungs during IAV infections in pigs or humans.

It was stated that the severity of the influenza disease is not crucial for bacterial co-infection, as an infection with mild symptoms already creates susceptibility to secondary infection (49). On the one hand, the pigs suffering from severe IAV infection had a poor prognosis due to high amounts of NETs, but on the other hand, the pigs with milder IAV disease were prone to bacterial co-infection. Apparently, in our study, several coinfected animals were identified (**Table S1**).

After identifying NETs in the BALF of IAV-positive diseased pigs *in vivo*, we investigated in the next step how the milieu with NETs in the lung of diseased pigs acts on the growth and survival of lung pathogenic bacteria that can be found in secondary infections. In previous studies, we reported that *Pasteurellaceae* show enhanced growth when they are incubated together with NET-releasing neutrophils (22, 23). With *in vitro* growth experiments in liquid culture, we are able to focus on the effects of an IAV infection, which is independent of the direct immunomodulation, e.g., inhibition of a distinct cell type. Differences in growth progression were detected: *A.pp* and *G. parasuis* profit stronger than *S. suis* from the presence of BALF in IAV-positive animals (**Figure 4**). By conducting a correlation analysis of markers detected in the BALF and the growth progression of *A.pp* in the presence of these BALF samples, we can narrow down which substances could potentially be responsible for promoting growth.

In general, the host releases DNase after NET formation to diminish overshooting NET formation and to clear them in later stages. Pathogens can release nucleases to evade NET entrapment (19, 50). In previous studies, we showed that *Pasteurellaceae* benefit from the degradation of NETs by DNases in another direction, as growth factors such as NAD become easily accessible (22, 23). However, in this study, DNase activity in the infected animals is significantly reduced compared with the healthy controls (**Figure 2E**). Enzymes have a distinct life span — a limited number of reactions they can catalyze before losing their function (51). The BALF samples used in our study were collected immediately after euthanasia in a late stage of the disease according to the lung tissue alterations. It is conceivable that DNase activity is reduced due to the effect of exhaustion, but detailed data about the DNase activity levels in the course of a respiratory infection are missing. Furthermore, the DNase activity does not correlate with the growth progression of *A.pp* (**Figure 5J**) in IAV-positive animals. In the IAV-negative animals, a tendency is detected that DNase activity in the BALF is positively correlating with the growth progression of *A.pp* (**Figure 5I**). This leads to the hypothesis that the growth in the BALF is influenced by several factors and not by only one factor.

Another factor that has been detected in the supernatant of NETs is NAD, the crucial growth factor of *Pasteurellaceae* (23). NAD was present in every sample, but no difference between the groups was detectable (**Figure 3A**). In 25 out of the 30 samples with a detected bacterial infection, *Pasteurellaceae* were present, so unneglectable amounts of NAD might have been used up during the infection. In fact, the four highest NAD values in IAV-positive BALF were from animals without a *Pasteurellaceae* infection (**Table S1**; **Figure 3A**). Comparing the levels of NAD in the BALF regarding the presence or absence of a *Pasteurellaceae* infection, a significantly lower amount of NAD in the *Pasteurellaceae*-positive samples was found (**Figure S1**). Although NAD was not the growth-enhancing key factor in the conducted *in vitro* assays (**Figures 5K, L**), it has to be taken into account *in vivo* that it could be a potential growth-enhancing factor in IAV-positive animals.

Furthermore, IAV infections increase the sialic acid content of BALF and lung tissue which can be utilized by pneumococci (52) and therefore are described as attractive nutrients for microbes (53). *A.pp* and *G. parasuis* are known to have sialic acid metabolizing genes (54, 55). Indeed, we detected significantly more sialic acid inside the BALF of IAV-positive animals. However, sialic acid was furthermore detected in some BALF samples from IAV-negative animals and did not correlate with the IAV Ct value (**Figures 3B, F**). One explanation for this finding could be other viral or bacterial infections as they are described to increase sialic acid as well, at least in the serum (56). The amount of sialic acid and the growth progression of *A.pp* correlated positively, and a more significant effect in the BALF of IAV-positive animals was detected (**Figures 5M, N**). In addition, a significant positive correlation between the growth progression of *S. suis* and the sialic acid content was found (**Figure S2**). This goes in line with the finding described in pneumococci (52).

In addition, several NET markers positively correlate with the growth progression of *A.pp* and are partially only significant (nucleosome, **Figure 5D**) or remarkable (H3Cit, **Figure 5H**) in IAV-positive animals. NETs are described for *A.pp* as one growth increasing factor (23). However, other lung infecting agents can induce NETs as well, and therefore, the observed growth increasing effect of BALF from IAV-negative animals is a conclusive consequence. Based on these findings, it can be hypothesized that not only one NET marker is explaining the growth increase of *A.pp* in the BALF of IAV-positive animals; rather, a mixture of several factors is potentially responsible. This seems to be the case in the growth progression of *G. parasuis* as well, as no correlation was identified (**Figure S3**). Further studies are needed to understand this complex *in-vivo* system.

Indeed, IAV may exhibit benefits on bacterial co-infections in several ways, independent of direct immunomodulatory effects, which could also benefit *S. suis*. Upregulation of the mucus and the destruction of cells provide nutrients for bacteria that could be also utilized *in vitro* (52).

It can be hypothesized that *in vivo* NAD from degraded NETs in a first step could enhance the growth of *Pasteurellaceae* during IAV infection. In later steps of a co-infection, other factors from NETs and destroyed lung tissue enhance the growth further, as this study observed NAD-independent growth enhancement by BALF of IAV-positive animals of bacteria. Other factors that can be released during IAV infection by lung tissue cells or other immune cells are interferons. We measured IFN-α and IFN-γ as two examples of interferons released during viral infections, but no clear effect on the growth of *A.pp*, *G. parasuis*, or *S. suis* was observed (**Figures 5O–R**, **S2O–R**, **S3O–R**). However, these results should not be interpreted too strongly as most of the samples used in the growth experiments had low to undetectable levels of interferon. After we have found the beneficial effects of BALF on bacteria, we aimed to investigate the effect that BALF has on the oxidative burst of neutrophils (**Figure 6**). ROS is one of the key effector functions of neutrophils and a powerful tool to control bacterial infections. An *in vivo* study in mice has shown that IAV decreased the activity of MPO to such an extent that it can be considered biologically irrelevant in the immune reaction (57). MPO produces the ROS hypochlorite from hydrogen peroxide and chloride and is important for the clearance of phagocytosed bacteria. Its deficiency is linked to an increase in severe infections (58). We detected significant reductions in the amount of ROS producing cells and in the intensity of the oxidative burst. In combination with bacterial virulence factors with antioxidant properties, e.g., SodA, SodC, and ohr of *A.pp* that give resistance against oxidative burst, the effectivity of ROS during co-infection may be severely reduced (59, 60). However, the inhibition in ROS could have more extensive effects than direct damage to pathogens, as MPO has beneficial effects on the activation and the survival of neutrophils via interaction with CD11b/CD18 (61).

Finally, the neutrophil killing assay aimed to investigate the killing behavior of neutrophils *in vitro*. In a previous study of our group, we found that *A.pp* is not killed by neutrophils and NETs but gains resources for their growth and the presence of DNase enhanced this growth boost (23). It is known that the Apx toxins and lipopolysaccharides (LPS) of *A.pp* sufficiently prevent phagocytosis and killing by neutrophils and result in the death of the immune cells (62). By the addition of BALF, we wanted to better mimic a more *in vivo*-like situation, as it provides neutrophils with cytokines and other factors of the immune system that are present in the infected lung. The BALF of IAV diseased pigs contains proinflammatory cytokines, e.g., IFN-α or IL-8 (63). IL-8 plays a key role in the recruitment and activation of neutrophils (64).

However, in our study, the ratios of the survival factors show that the BALF of an IAV-positive pig is significantly benefiting the survival of *A.pp* in the presence of neutrophils. Which factors exactly are responsible is not yet fully clarified. The IAV-positive BALF sample used in this experiment had a substantially less amount of NAD than the IAV-negative BALF sample (IAV-positive: 136.42 ng/ml; IAV-negative: 391.60 ng/ml). Therefore, the NAD does not cause the effect of the BALF on the survival of *A.pp*. In contrast to this, the amount of cell-free DNA is twice as high (IAV-positive: 310.73 ng/ml; IAV-negative: 158.62 ng/ml) and could serve nutrients to *A.pp* after degradation (23, 65). DNase activity in the BALF was measured in both animals included in this assay, so both pre-existing and freshly induced NETs get degraded and provide nutrients to *A.pp* DNase additionally allows to recycle DNA of deceased bacterial cells. *A.pp* does not have its own extracellular DNase that is able to degrade NETs, but it profits from the NET-degrading DNases of the host or coinfecting bacteria. Bacteria that were detected in this project and produced a DNase included *S. suis* (SsnA, EndAsuis), *G. parasuis* (CdtB), *Salmonella typhimurium*, and *Mycoplasma hyorhinis* (22, 48, 66, 67). Also, the previously mentioned increase in sialic acid in the lung caused by IAV could have played a role (52). Ultimately, it is also conceivable that the substances, which would benefit the neutrophils, became too diluted during the lavage, so a potential effect is not strong enough anymore.

## 5 Conclusion

In conclusion, our study presents for the first time evidence for vesicular NET formation in pigs during IAV infection. Several NET markers are present in the BALF of IAV-positive pigs and correlate with the viral load. The milieu in the BALF of IAV diseased pigs promotes the growth of lung pathogenic bacteria like *A.pp* and *G. parasuis* and inhibits oxidative burst in porcine neutrophils. Thus, factors from the BALF do not facilitate neutrophils to control an infection with *A.pp in vitro*. This reveals another weak spot of the host’s immune system during IAV infection that may contribute to the severity of bacterial co-infection. However, one limitation of this study is that not one factor was identified that can explain the phenotypes. It is only possible to conclude that a mixture of different factors from NETs, sialic acid, and further potentially unknown factors is growth enhancing for bacteria like *A.pp* in the BALF of IAV-positive animals. Nevertheless, this study underlines how complex the *in vivo* situation is during a co-infection. Even though the *in vivo* situation is complex and no clear black-and-white picture exists, it was possible to identify differences between IAV-positive and IAV-negative animals regarding the influence of the lung milieu on co-infections. Therefore, especially if IAV induces high amounts of NETs and sialic acid, this is a potential growth enhancing mixture of factors. The data suggest that the higher the IAV load, the higher the NET marker levels and the higher the growth-promoting effect for coinfecting bacteria by NETs and sialic acid. Nevertheless, further studies are needed to better understand the host–pathogen interaction during co-infections and thereby identify new possibilities to prevent co-infections during IAV infection.

It can be hypothesized that comparable situations can be observed with human BALF from IAV-infected patients and *Haemophilus influenzae*. Therefore, first, this study contributes to the understanding of ongoing co-infections in pigs and, second, may serve to translate these new findings to humans in future studies.

## Data availability statement

The original contributions presented in the study are included in the article/**Supplementary Material**. Further inquiries can be directed to the corresponding author.

## Ethics statement

The handling and treatment of all animals were conducted in strict accordance with the principles outlined in the EU Directive 2010/63/EU and the German Animal Protection Law (Tierschutzgesetz). The blood sampling was approved by the authorities in the Committee on Animal Experiments of the Lower Saxonian State Office for Consumer Protection and Food Safety [Niedersáchsisches Landesamt fúr Verbraucherschutz und Lebensmittelsicherheit (LAVES)], Lower Saxony, Germany, under the registration number: 33.9-42502-05-18A302. Written informed consent was obtained from the owners for the participation of their animals in this study.

The collection of BALF from diseased pigs sent for routine diagnostics was conducted in the Field Station for Epidemiology, University of Veterinary Medicine Hannover, Bakum, Germany. Pigs from swine farms in Lower Saxony, Germany, with visible symptoms of respiratory disease were euthanized for routine necropsy. The euthanasia was carried out according to the belowdescribed “Guideline for the implementation of emergency killing of pigs.” The euthanasia of pigs due to other reasons, to collect BALF of healthy pigs, was approved and registered by the local Animal Welfare Officer in accordance with the German Animal Welfare Law under number TiHo-T-2019-14.

## Author contributions

SL: Data curation, Formal analysis, Investigation, Methodology, Validation, Visualization, Writing – original draft, Writing – review & editing. IH-P: Investigation, Resources, Writing – review & editing. MB: Investigation, Writing – review & editing. MM: Investigation, Visualization, Writing – review & editing. RI: Investigation, Writing – review & editing. MK-B: Supervision, Writing – review & editing. NB: Conceptualization, Data curation, Formal analysis, Funding acquisition, Investigation, Methodology, Project administration, Supervision, Validation, Visualization, Writing – original draft, Writing – review & editing.
