# Supplementary material for: Impact of bronchoalveolar lavage from influenza A virus diseased pigs on neutrophil functions and growth of co-infecting pathogenic bacteria
Source: Front Immunol. 2024 Feb 21;15:1325269. doi: 10.3389/fimmu.2024.1325269 (PMC10914936; doi:10.3389/fimmu.2024.1325269)
Supplement: Supplementary file 1 [file DataSheet_1.pdf]

Supplementary Material

1 Supplementary Figures and Tables

1.1 Supplementary Figures

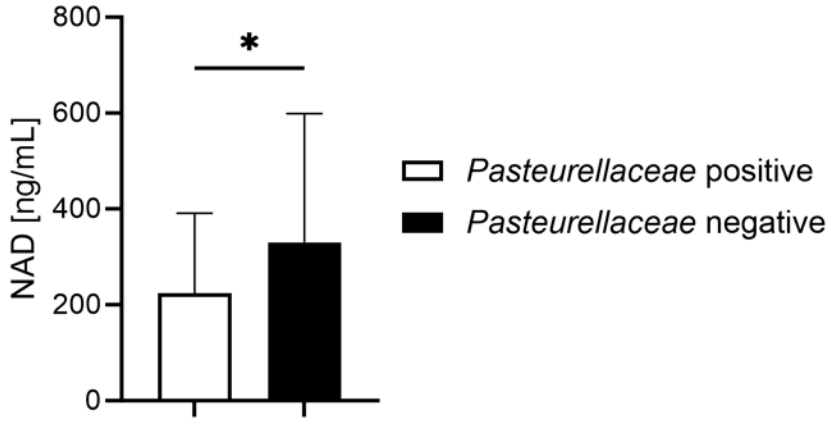

Supplementary figure 1: NAD levels in BALF sorted by *Pasteurellaceae* infection

The results of the nicotineamide adenine dinucleotide (NAD) assay shown in **Fig. 3A** in an alternative presentation sorted by the presence of *Pasteurellaceae* bacteria, as depicted in **Table S1**. *Pasteurellaceae* positive n=25, *Pasteurellaceae* negative n=20. A Mann-Whitney-test was calculated (*p<0.05).

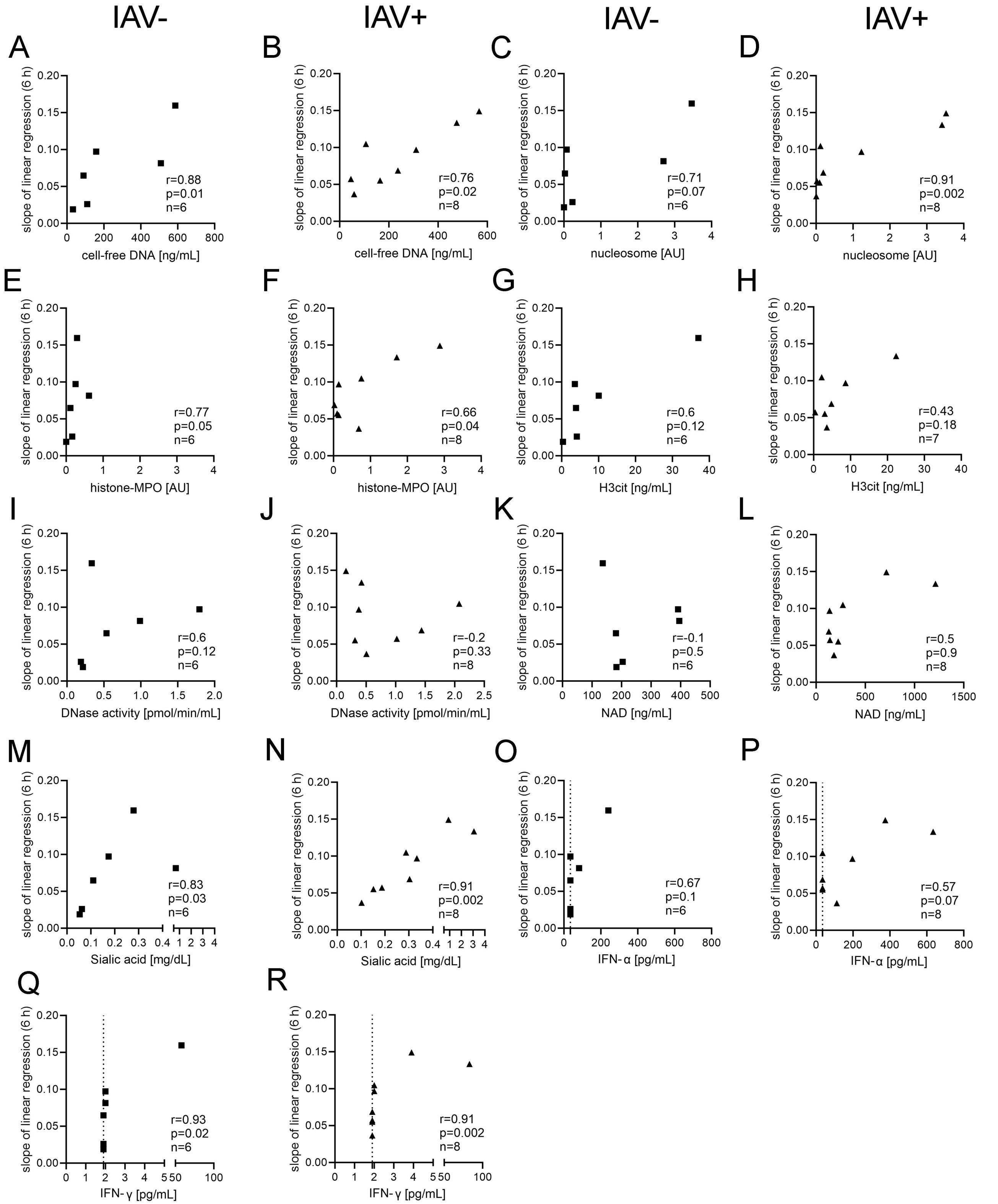

Supplementary figure 2: Correlation analysis of NET markers and other markers detected in bronchoalveolar lavage fluid (BALF) with the growth progression of *S. suis* in BALF. The correlation analysis was performed with Spearman *r* for IAV-negative BALF (**A, C, E, G, I, K, M, O** and **Q**) and for IAV-positive BALF (**B, D, F, H, J, L, N, P** and **R**) and detected several significant

positive correlations. The growth progression of *S. suis* correlates positive with cell-free DNA (**A** and **B**), histone-MPO (**E** and F), sialic acid (**M** and **N**) and IFN-γ (**Q** and **R**) in case of IAV-negative and IAV-positive BALF. The growth progression of *S. suis* correlates with nucleosome (**D**) only in case of IAV-positive BALF. The values for *r*, *p* and n are given in each graph and each square or triangle represent one BALF sample.

Several markers in BALF correlate positive with the growth progression of *S. suis*, but this was not significant. The dotted line (**O-R**) represents the detection limit of the ELISA for IFN-α (36 pg/mL) and IFN-γ (2 pg/mL), as all samples were no signal was detected were set to the detection limit.

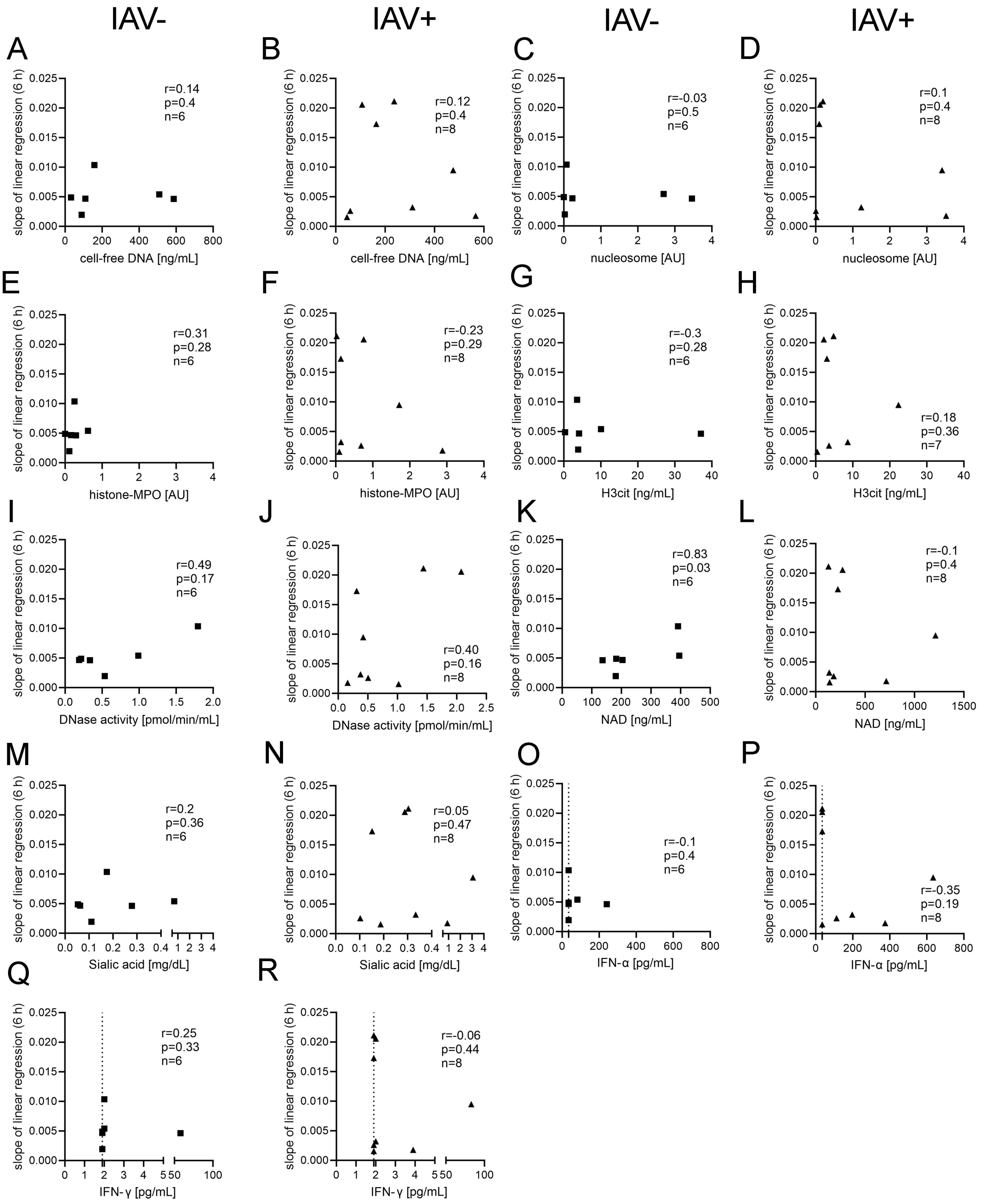

Supplementary figure 3: Correlation analysis of NET markers and other markers detected in bronchoalveolar lavage fluid (BALF) with the growth progression of *G. parasuis* in BALF. The correlation analysis was performed with Spearman *r* for IAV-negative BALF (**A, C, E, G, I, K, M, O** and **Q**) and for IAV-positive BALF (**B, D, F, H, J, L, N, P** and **R**) and detected no significant correlations except one. The growth progression of *G. parasuis* correlates positive with NAD) in case

of IAV-negative BALF (**K**). The values for *r*, *p* and n are given in each graph and each square or triangle represent one BALF sample. The dotted line (**O-R**) represents the detection limit of the ELISA for IFN-α (36 pg/mL) and IFN-γ (2 pg/mL), as all samples were no signal was detected were set to the detection limit.

1.2 Supplementary Tables

Table S1: Overview about pigs that were included in this study

number	sex	age	weight [kg]	pathogenic bacteria in bronchi	pathogenic bacteria in lung	ct-value IAV lung	ct-value IAV BALF	other viruses
**1**	f	Weaner	8,7	(-)	(-)	29	28	(-)
**2**	m-	Porker	22	*G. parasuis* +++	(-)	(-)	(-)	(-)
**3**	f	Porker	65	(-)	*A. pleuropneumoniae* +++	(-)	(-)	(-)
**4**	f	Weaner	8,1	*G. parasuis* +++<br>*S. suis* +	*G. parasuis* +++<br>*S. suis* +	14	17	(-)
**5**	m-	Weaner	6,9	*G. parasuis* +++<br>*S. suis* +	*G. parasuis* +++<br>*S. suis* +++	20	19	(-)
**6**	m-	Weaner	8,7	*G. parasuis* +++	*G. parasuis* Serotype 7 +++	(-)	22	(-)
**7**	f	Weaner	7,6	*G. parasuis* +	*G. parasuis* +++	(-)	26	(-)
**8**	f		40	(-)	(-)	(-)	(-)	(-)
**9**	f	Sow	223	*P. multocida* ++	*P. multocida* +	(-)	(-)	(-)
**10**	f	Weaner	6,1	*G. parasuis* +	(-)	(-)	(-)	(-)
**11**	f	Weaner	7,8	*G. parasuis* +++	(-)	(-)	N/A	(-)
**12**	f	Weaner	14,2	(-)	(-)	31	32	(-)
**14**	f	Weaner	17,7	(-)	(-)	32	33	(-)
**13**	m-	Weaner	14,7	(-)	(-)	36	31	(-)
**15**	m-	Weaner	13	*B. bronchiseptica* +++	(-)	31	31	PCV2
**16**	f	Weaner	16,9	(-)	(-)	(-)	(-)	(-)
**17**	m-	Weaner	16,7	*S. typhimurium* +++<br>*G. parasuis* +++<br>*S. suis* +++	*S. typhimurium* +++<br>*S. suis* +++	(-)	N/A	(-)
**18**	m-	Weaner	20	*M.hyorhinis* ++	N/A	(-)	(-)	PRRSV EU
**19**	m-	Weaner	8,6	*G. parasuis* +++	*G. parasuis* +++	(-)	(-)	(-)
**20**	m-	Weaner	7,6	*G. parasuis* +++	*G. parasuis* +++	(-)	(-)	(-)
**21**	m-	Weaner	5,81	*G. parasuis* +	*M. hyorhinis*	(-)	(-)	(-)
**22**	m-	Weaner	5,79	*G. parasuis* +++	(-)	(-)	(-)	(-)
**23**	m-	Weaner	6,8	*G. parasuis* ++	*G. parasuis* Serotype 5/12 +	(-)	(-)	PRRSV
**24**	m-	Weaner	11	(-)	(-)	(-)	(-)	(-)
**25**	f	Weaner	16	(-)	(-)	(-)	(-)	PRRSV
**26**	m-	Weaner	11,9	*G. parasuis* Serotype 1 +++	(-)	(-)	(-)	(-)
**27**	m-	Weaner	10,9	*G. parasuis* Serotype 1 +++	*G. parasuis* Serotype 1 +	(-)	(-)	(-)
**28**	f	Weaner	9	(-)	(-)	(-)	(-)	(-)
**30**	f	Pig	29	*G. parasuis* +	(-)	(-)	25	(-)
**31**	m-	Weaner	6,3	(-)	(-)	(-)	27	(-)
**32**	f	Weaner	4	*S.suis* +	*S. suis* +	(-)	30	(-)
**33**	f	Sow	4,5	(-)	*G. parasuis* +++	34	(-)	(-)
**34**	m-	Sow	5,6	N/A	(-)	(-)	20	(-)
**35**	f	Weaner	6,3	*G. parasuis* +++<br>*S. suis*+	*G. parasuis* +++<br>*S. suis*+	31	(-)	(-)
**36**	f	Weaner	6	N/A	(-)	(-)	(-)	(-)
**37**	m-	Weaner	7,5	*G. parasuis* +++<br>*S. suis.*+++	*G. parasuis* +++<br>*S. suis* ++	26	(-)	(-)
**38**	f	Gilt	121	N/A	(-)	26	(-)	(-)
**39**	f	Gilt	134	(-)	(-)	25	(-)	(-)
**40**	m-	Sow	3,1	(-)	(-)	(-)	29	(-)
**41**	m-	Sow	3,2	*G. parasuis* +++	*G. parasuis* +	(-)	(-)	(-)
**42**	f	Sow	2,6	*B. bronchiseptica* +++<br>*G. parasuis* +++	(-)	(-)	(-)	(-)
**43**	f	Sow	289	(-)	β-hem *Streptococcus spp.* +	37	32	(-)
**44**	f	Sow	239	N/A, overgrown with<br>*Enterobactericae*	(-)	24	23	(-)
**45**	m-	Pig	50	*A. pleuropneumoniae* +++	*A. pleuropneumoniae* Serotype 2 +++	(-)	(-)	PRRSV
**46**	m-	Pig	112	*A. pleuropneumoniae* +++<br>β-hem *Streptococcus spp.* +++	*A. pleuropneumoniae* Serotype 5 +++<br>β-hem *Streptococcus spp.* +++	(-)	(-)	(-)
**T13**	f	Porker	50	(-)	(-)	(-)	(-)	(-)
**T14**	m-	Porker	90	(-)	(-)	(-)	(-)	(-)
**T15**	m-	Porker	90	(-)	(-)	(-)	(-)	(-)
**T16**	m-	Porker	90	(-)	(-)	(-)	(-)	(-)
**Br**	f	Weaner	18	(-)	(-)	(-)	(-)	(-)

Abbreviations

f = female; m= male; - = castrated; (-) = not detected; + = minor infestation; ++ = moderate infestation; +++ = severe infestation; *G.* = *Glaesserella* ; *S.* = *Streptococcus* ; *P* . = *Pasteurella* ; *B.* = *Bordetella* ; *S.* = *Salmonella* ; *M.* = *Mycoplasma* ; N/A = not available; *A.* = *Actinobacillus* ; β-hem = beta-hemolytic; *spp.* = *species pluralis* ; PCV2 = porcine circovirus type 2; PRRSV EU = porcine reproductive and respiratory sydrome virus european type
